# A universal co‐expression gene network and prognostic model for hepatic–biliary–pancreatic cancers identified by integrative analyses

**DOI:** 10.1002/2211-5463.13478

**Published:** 2022-09-23

**Authors:** Jing Zhang, Juan Xiao, Yixuan Wang, Xiao Zheng, Jiajun Cui, Chaochen Wang

**Affiliations:** ^1^ Zhejiang University‐University of Edinburgh Institute (ZJU‐UoE Institute), Zhejiang University School of Medicine, International Campus, Zhejiang University Haining China; ^2^ Guangxi Key Laboratory of Molecular Medicine in Liver Injury and Repair Affiliated Hospital of Guilin Medical University China

**Keywords:** cholangiocarcinoma, hepatocellular carcinoma, pancreatic adenocarcinoma, prognosis, training cohort, validation cohort

## Abstract

Hepatic, biliary and pancreatic cancers are a diverse set of malignancies with poor prognoses. It is possible that common molecular mechanisms are involved in the carcinogenesis of these cancers. Here, we identified LINC01537 and seven protein‐coding genes by integrative analysis of transcriptomes of mRNAs, microRNAs and long non‐coding RNAs from cholangiocarcinoma, hepatocellular carcinoma and pancreatic adenocarcinoma cohorts in TCGA. A predictive model constructed from seven biomarkers was established to successfully predict the survival rate of patients, which was then further verified in external cohorts. Additionally, patients with high‐risk scores in our model were prone to epithelial–mesenchymal transition. Finally, activation of the biomarker PDE2A significantly attenuated migration and epithelial–mesenchymal transition in the HepG2 liver cancer cell line.

## Graphical abstract

Hepatic, biliary and pancreatic cancers are a diverse set of malignancies with poor prognoses. Here, we established a prognostic model for hepatic–biliary–pancreatic cancers based on *ACSM5, ADRA1A, CCL14, DNASE1L3, LILRB5, PDE2A* and *RBP5* expression. Our prognostic model was predictive of patient survival in both TCGA test cohorts and in two independent validation cohorts.

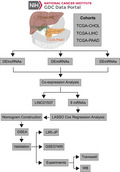

**Abbreviations**
ceRNA: competitive endogenous RNA
CHOL: cholangiocarcinoma
DElncRNA: differentially expressed lncRNA
DEmRNA: differentially expressed mRNA
DEmiRNA: differentially expressed miRNA
EMT: epithelial–mesenchymal transition
GSEA: Gene Set Enrichment Analysis
ICGC: International Cancer Genome Consortium
LASSO: least absolute shrinkage and selection operator
lncRNA: long non‐coding RNA
LIHC: liver hepatocellular carcinoma
miRNA: micro RNA
miRNA‐seq: microRNA‐sequencing
OS: overall survival
PAAD: pancreatic adenocarcinoma
RNA‐seq: RNA‐sequencing
sgRNA: single guide RNA
TCGA: The Cancer Genome Atlas

The digestive system consists of the alimentary canal and accessory organs [1]. The liver and pancreas, the main accessory organs of the digestive system, provide various enzymes for metabolism in living organisms [2]. Bile ducts are responsible for transporting bile, produced by hepatocytes, to the small intestine at the duodenum for its biological functions [2]. The hepatic–biliary–pancreatic system relies on a small endoderm progenitor compartment that gives rise to a variety of different mature tissues and organs, including the liver, pancreas, gall bladder and extra‐hepatic bile ducts [3]. Malignances regarding to these three important digestive gland organs have become prevalent in recent years, with a relatively poor prognosis.

Primary liver cancer, including hepatocellular carcinoma (75–85% of cases) and intrahepatic cholangiocarcinoma (10–15% of cases), has become the sixth most commonly cancer and the fourth leading cause of cancer death worldwide [4]. The 5‐year survival rate is only 8.9% for hepatocellular carcinoma and 8% for cholangiocarcinoma [5, 6]. Pancreatic adenocarcinoma is another devastating malignancy with a 1‐year survival rate of approximately 18%, and a 5‐year survival rate of < 4% [7, 8]. Although therapeutic strategies have largely improved in recent years, prognosis remains unsatisfactory. Comprising important digestive glands associated with metabolism, the liver, bile ducts and pancreas are closely interrelated in organ development, disease progression and the mechanism of tumors [9, 10]. Therefore, we inferred that there might be a common mechanism underlying the malignancies of the three organs, which has been ignored in previous studies. In the present study, we aimed to elucidate the common mechanisms of tumorigenicity and identifying novel biomarkers in the above digestive gland malignancies.

Recently, accumulating evidence has verified that mRNAs, microRNAs (miRNAs) and long non‐coding RNAs (lncRNAs) participated in the initiation and progression of several tumor types, including hepatocellular carcinoma, cholangiocarcinoma and pancreatic adenocarcinoma [11, 12, 13]. miRNAs are small non‐coding RNAs (ncRNA) that repress gene expression by binding to 3′ untranslated region of complementary mRNA sequence through miRNA response elements to negatively regulate target mRNAs translation or promote mRNA degradation and silencing [14]. Additionally, the regulatory functions of miRNA can be diluted by competition from other molecules, called competitive endogenous RNAs (ceRNAs). This hypothesis was first proposed by Salmena et al. [15], indicating that ncRNAs can compete with miRNA via acting as natural miRNA sponges by virtue of sharing miRNA response elements. lncRNAs are defined as RNA transcripts longer than 200 nucleotides [16]. lncRNAs interact with proteins, DNAs and mRNAs to form complex intramolecular and intermolecular secondary and high‐order structures through diverse molecular mechanisms, thus mediating downstream gene expression, regulating protein activity, controlling alternative mRNAs splicing and providing scaffolding for chromatin modification [17]. lncRNAs can also function as ceRNAs by competitively binding to miRNAs, and thereby indirectly regulate tumor‐related gene expression at transcriptional or post‐transcriptional levels [18]. Therefore, mRNAs, miRNAs and lncRNAs synergistically participate in diverse cancer biological processes, such as proliferation, apoptosis, metastasis, migration, angiogenesis, metabolism and drug resistance [11, 12, 13, 19]. However, whether a common molecule network exists in hepatic, biliary and pancreatic cancers is unknown.

Genome‐wide transcriptomic studies have been performed in various cancers and provide an opportunity to systematically study the molecular mechanisms across different types of cancers [20]. Here, we obtained the genome‐wide expression profiles of lncRNAs, miRNAs and mRNAs and their corresponding clinical information of patients in cholangiocarcinoma (CHOL), liver hepatocellular carcinoma (LIHC) and pancreatic adenocarcinoma (PAAD) cohorts from The Cancer Genome Atlas (TCGA) data portal. By integrative analysis, we discovered a lncRNA LINC01537 with a potential role in three types of digestive gland cancers and established a robust prognosis‐associated prediction model composed of seven protein‐coding genes. This model effectively predicted patient survival status in both internal test cohort and external validation cohorts. The samples with high‐risk score in our model highly express the signature of epithelial–mesenchymal transition (EMT), suggesting that genes in our model might be associated with EMT. Consistently, activation of PDE2A, one of genes in this model, reversed the expression of EMT signatures and repressed the cell invasion in hepatocellular carcinoma cells.

## Materials and methods

### Data filtration

Systematic searches of the Cancer Genome Atlas (TCGA) database for the CHOL, LIHC and PAAD cohorts were performed. Level 3 RNA‐sequencing (RNA‐seq) count data (mRNA and lncRNA; Illumina HiSeq RNA‐seq platform; Illumina, San Diego, CA, USA) and miRNA‐sequencing (miRNA‐seq) count data (Illumina HiSeq miRNA‐Seq platform), as well as their corresponding clinical phenotypic data and survival information, were obtained from the GDC Data Portal (https://portal.gdc.cancer.gov) [21]. For mRNA expression, 45 samples (nine normal samples and 36 tumor samples) in the CHOL cohort, 424 patients in the LIHC cohort (50 normal samples and 374 tumor samples) and 182 samples in the PAAD cohort (covering four normal samples and 178 tumor samples) were included. Meanwhile, the miRNA expression data of CHOL, LIHC and PAAD were gathered from 45 samples (nine normal samples and 36 tumor samples), 425 patients (50 normal samples and 375 tumor samples) and 183 samples (four normal samples and 179 tumor samples), respectively. In this study, patients with both mRNA‐seq expression data and miRNA‐seq expression data in each cohort were selected for subsequent analysis. After data filtration, LIHC expression data of 416 patients were retained, consisting 49 normal samples and 367 tumor samples. PAAD expression data were retained in 182 patients, including four normal samples and 178 tumor samples. All patients in the CHOL cohort were eligible and no data filtration was required.

Essential clinical information consisting of patient ID, gender, age, TNM staging, tumor histological grading, survival state and survival time was also extracted. We excluded cases from TCGA database according to the following criteria: (a) patients without survival statues and survival time information; (b) patients without their corresponding mRNA or miRNA expression data; (c) patients with a follow‐up time < 1 month. In total, 469 patients were selected in this project.

The mutation data for CHOL cohort, LIHC cohort and PAAD cohort were also downloaded from the GDC Data Portal (https://portal.gdc.cancer.gov) [21].

Additionally, the LIRI‐JP cohort downloaded from the International Cancer Genome Consortium (ICGC; https://dcc.icgc.org) and the data from GSE57495 [22] in GEO database (https://www.ncbi.nlm.nih.gov/geo/query/acc.cgi?acc=GSE57495) were used as the external validation cohorts to further test the efficacy of the predictive accuracy of prognostic model.

Finally, the data from GSE5203 [23] in GEO database (https://www.ncbi.nlm.nih.gov/geo/query/acc.cgi?acc=GSE5203) were utilized for subsequent experimental validation. All of the data were publicly available and there were no ethical issues involved.

### Differential expression analysis

The deseq2 package [24] in bioconductor was used to reveal the differentially expressed mRNAs (DEmRNAs), differentially expressed miRNAs (DEmiRNAs) and differentially expressed lncRNAs (DElncRNAs). The significant different thresholds are *P* < 0.05 and ¦log2 fold change ¦ ≥ 1. The GENCODE database was used for distinguish lncRNAs from mRNAs (https://www.gencodegenes.org). Simultaneously, the ‘GDC.h38 GENCODE v22 GTF’ file was download from GDC to annotate mRNA and lncRNA from RNA‐seq. Venn diagrams were performed to visualize the uniformly upregulated or downregulated DEmRNAs, DEmiRNAs and DElncRNAs.

### Functional enrichment and pathway analysis

Gene Set Enrichment Analysis (GSEA) against hallmark gene sets in MsigDb [25] was performed using genes ranked by log2‐transformed fold change (log2FC) with the fgsea package [26] in r. To evaluate the potential functions of uniformly upregulated or downregulated genes of the cancers, Gene Ontology (GO; http://geneontology.org) and Kyoto Encyclopedia of Genes and Genomes (KEGG; https://www.genome.jp/kegg) pathway enrichment analysis was performed using the clusterprofiler package in r [27]. The significant threshold value was set as *P* < 0.05. To explore the significantly enriched gene sets between high‐risk and low‐risk groups, GSEA analysis was also performed as described above.

### Co‐expression analysis

Co‐expression analysis was implemented using the Pearson correlation coefficient. Because co‐expression is one of the features of the ceRNA network, lncRNA–miRNA and miRNA–mRNA pairs with *P* < 0.05 and *r* ≤ −0.3 in each cohort were selected for ceRNA construction [28, 29]. Next, the intersections of co‐expressed lncRNAs–miRNAs and miRNAs–mRNAs in digestive gland malignancies were obtained. Finally, the lncRNA, miRNA and mRNA co‐expression network was established and visualized using the ggalluvial r package [30].

### Survival analysis

The relationship between mRNA expression level and overall survival (OS) of patients was evaluated by univariate Cox regression analysis. mRNAs with *P* < 0.05 were selected for further analysis. Furthermore, Kaplan–Meier survival analysis of DEmRNAs and DElncRNA was performed using the survival package in r [31]. Patients were separated into high‐risk and low‐risk groups according to gene median value. The *P* value was calculated utilizing a log‐rank test and DEmRNAs and DElncRNA with *P* < 0.05 were considered statistically significant.

### Risk score calculation

A risk score signature was constructed utilizing the most robust markers, which were selected by least absolute shrinkage and selection operator (LASSO) Cox regression. The risk score signature was then calculated using:

$$\text{Risk score}=\sum_{i=1}^{n}\text{Coef}i*\mathrm{Exp}i$$

where *n*, Coef and Exp are the number of signature genes, the coefficient obtained from LASSO Cox regression and the expression of signature genes, respectively. Features, consisting of gender, age, TNM grading and tumor histological staging, were submitted and analyzed by multivariate Cox regression to determine factors related with prognosis. *P* < 0.05 was set as the threshold to identify independent prognostic factors.

### Establishment of nomogram model for survival rate prediction

A nomogram model of independent factors was constructed and visualized using the regplot package in r [32]. To verify the predictive ability of the nomogram, the concordance index (C‐index) of the independent prognostic factors and the fitting coxph model, consisting of pathologic T and risk group, were calculated.

### Single guide RNA (sgRNA) design and plasmid construction

sgRNAs targeting PDE2A promoter region were designed by publicly available online tools (https://zlab.bio/guide‐design‐resources). Synthesized sgRNA oligonucleotides were annealed, phosphorylated and cloned into the digested plenti‐sg‐mCherry vector. The sgRNA sequences targeted PDE2A are shown in Table 1.

**Table 1:** sgRNA sequences.

Genes	Forward primer (5′ to 3′)	Reverse primer (5′ to 3′)
PDE2A‐sg1	caccGTGGGGTCGGAGGATCCGAC	aaacGTCGGATCCTCCGACCCCAC
PDE2A‐sg2	caccAGACAGAAGCGGGGTGACAG	aaacCTGTCACCCCGCTTCTGTCT

### Cell culture and transfection

HepG2 cells were obtained from the Cell Bank of Chinese Academy of Sciences (Shanghai, China) and cultured in Dulbecco's modified Eagle's medium (BI, Cromwell, CT, USA) containing 10% fetal bovine serum (BI), 1% penicillin/streptomycin (BI) at 37 °C in 5% CO_2_. All cell lines were tested for mycoplasma contamination.

plenti‐sg‐mCherry‐vector, plenti‐sg‐mCherry‐PDE2A‐sg1 and plenti‐sg‐mCherry‐PDE2A‐sg2 were respectively co‐transfected with dCas9‐VP64‐puro (plasmid #99371; Addgene, Watertown, MA, USA) utilizing Lipofectamine 3000 (Invitrogen, Waltham, MA, USA). After 24 or 48 h, cells were collected for subsequent experiments.

### Quantitative RT‐PCR

Total RNA was extracted using Trizol reagent (Invitrogen) in accordance with the manufacturer's instructions. Two micrograms of RNA was used for cDNA synthesis (Abm, Richmond, BC, Canada). qPCR was performed using EvaGreen 2xqPCR MasterMix (Abm) and CFX96 Real‐time PCR Detection System (Bio‐Rad, Hercules, CA, USA). Each sample was determined with triplicate independent experiments. The relative target gene expression, as a fold change above control group after normalization to GAPDH, was calculated using the 2^−ΔΔCt^ method. Primers used for amplification are shown in Table 2.

**Table 2:** Primers for PCR.

Genes	Forward primer (5′ to 3′)	Reverse primer (5′ to 3′)
GAPDH	CTGGGCTACACTGAGCACC	AAGTGGTCGTTGAGGGCAATG
PDE2A	GACCGCAAGATCCTCCAACTG	CCGAGCACTTTGTCTCCGA

### Transwell assay

Cells (2 × 10^5^) were resuspended in 200 μL of fetal bovine serum‐free medium and placed in the upper chambers (Corning Inc., Corning, NY, USA). Then, 600 μL of medium supplemented with 10% fetal bovine serum was added to the lower chambers. After 48 h of incubation at 37 °C, the cells were fixed with 4% paraformaldehyde (Solarbio, Beijing, China), stained with 0.1% crystal violet (Solarbio) and imaged.

### Western blotting

Western blot was performed using antibodies against β‐actin (ZSGB‐BIO, Beijing, China), E‐cadherin, N‐cadherin and Snail (Epithelial–Mesenchymal Transition Antibody Sampler Kit, # 9782T; Cell Signaling Technology, Danvers, MA, USA) with 1 : 1000 dilutions.

### Statistical analysis

Statistical analyses were performed using r, version 4.0.3 (R Foundation, Vienna, Austria) and prism, version 8 (GraphPad Software Inc., San Diego, CA, USA). All data are presented as the mean ± SD. Pairwise comparisons were performed based on a two‐tailed Student's *t*‐test. *P* < 0.05 was considered statistically significant.

## Results

### Differentially expressed genes in CHOL, LIHC and PAAD

**Fig. 1:**
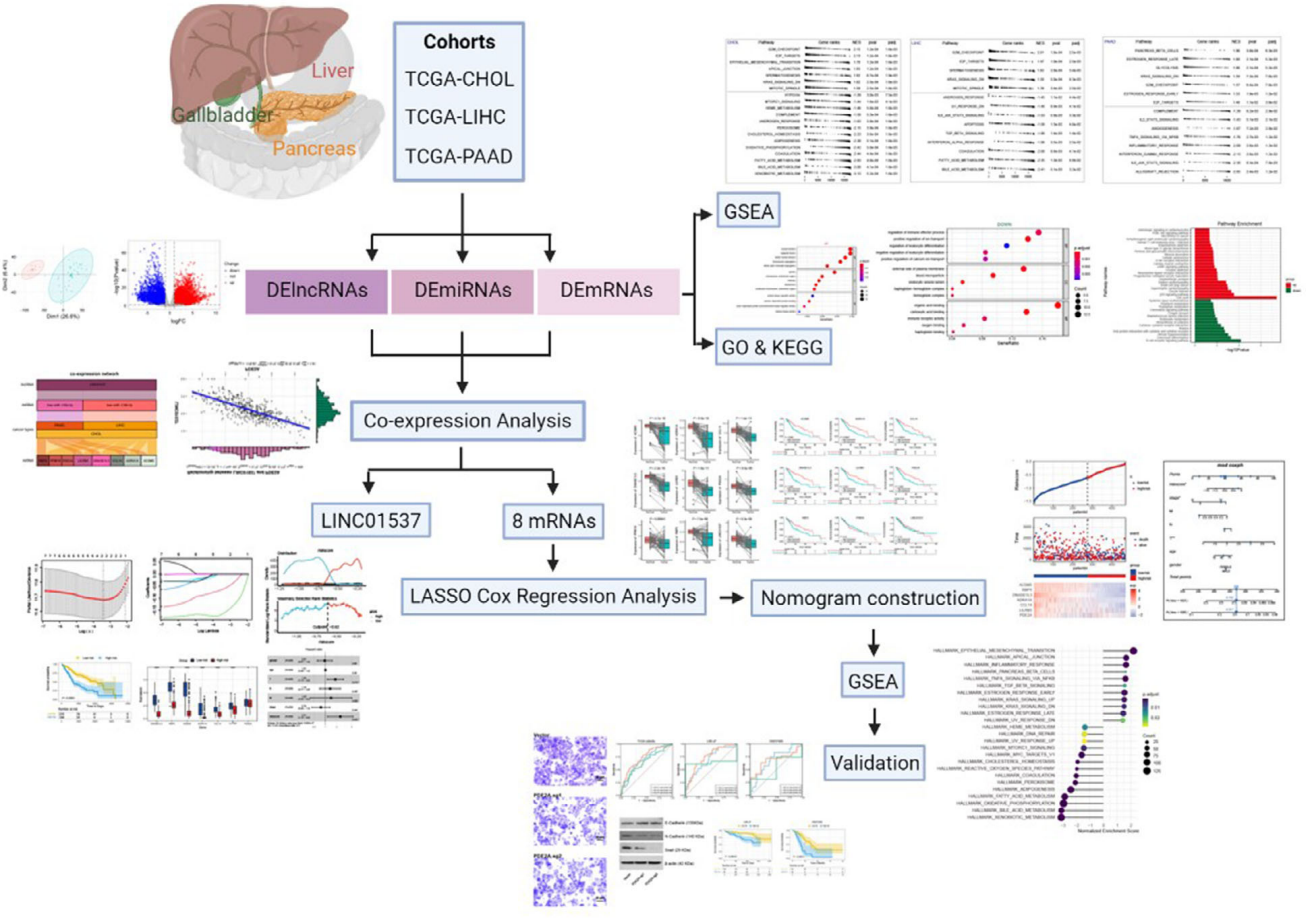
Schematic flowchart for the study. CHOL, cholangiocarcinoma; DElncRNA, differentially expressed long non‐coding RNA; DEmiRNA, differentially expressed microRNA; DEmRNA, differentially expressed mRNA; GSEA, gene set enrichment analysis; LASSO, least absolute shrinkage and selection operator analysis; LIHC, liver hepatocellular carcinoma; PAAD, pancreatic adenocarcinoma.

**Fig. 2:**
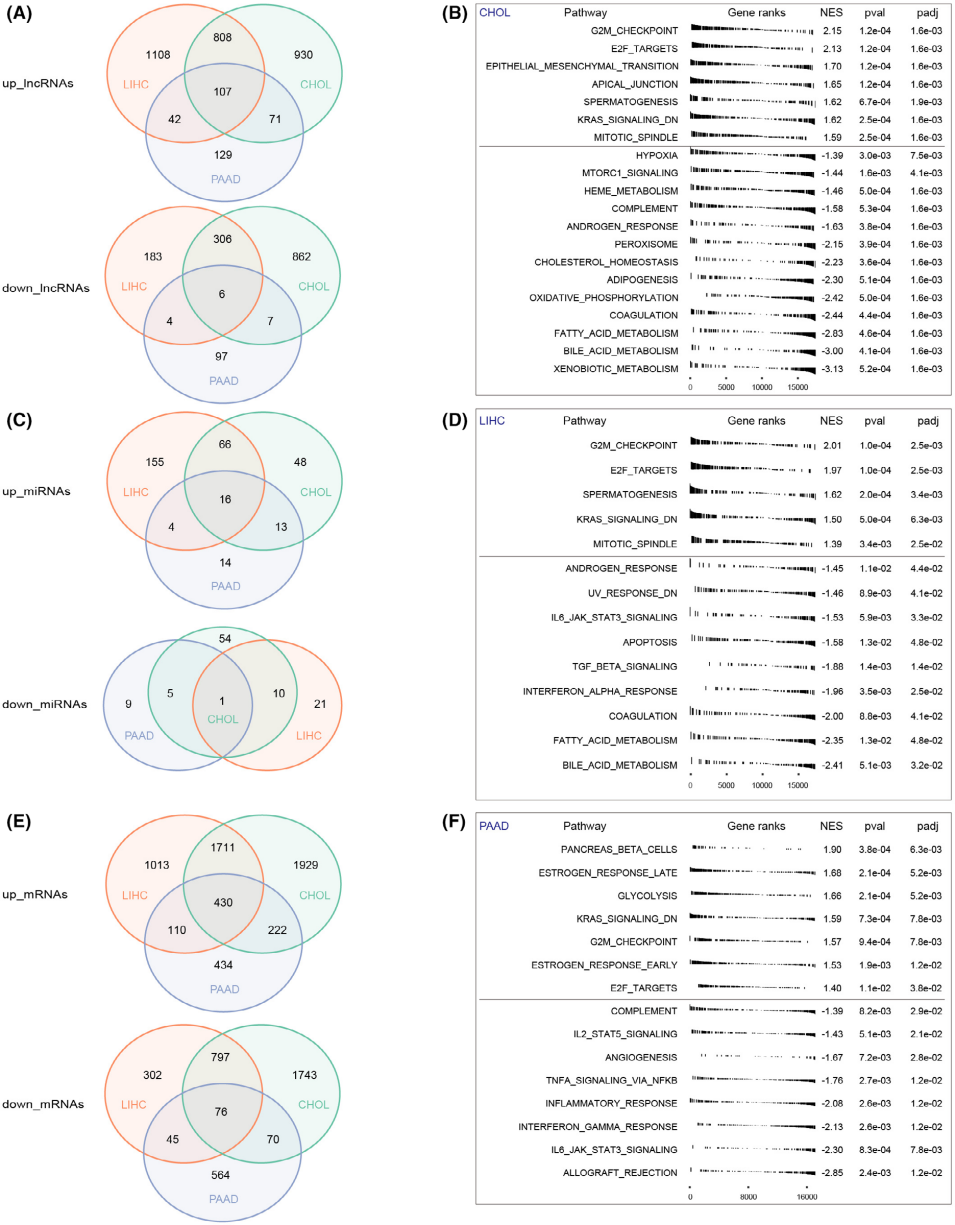
Venn diagram of overlapped lncRNAs, miRNAs and mRNAs and the hallmarks of different malignancies. The overlapped aberrantly expressed up‐regulated and down‐regulated (A) lncRNAs, (C) miRNAs and (E) mRNAs. Green circle, CHOL cohort; orange circle, LIHC cohort; purple circle, PAAD cohort. The hallmarks of (B) CHOL cohort, (D) LIHC cohort and (F) PAAD cohort.

A brief overview of the screening strategy employed in the present study is shown in Fig. 1. To identify genes that are involved all three cancers, differentially expressed mRNAs, miRNAs and lncRNAs were first characterized. In CHOL, 6978 DEmRNAs (4292 up‐regulated and 2686 down‐regulated), 213 DEmiRNAs (143 up‐regulated and 70 down‐regulated) and 3097 DElncRNAs (1916 up‐regulated and 1181 down‐regulated) were presented (Fig. S1). In LIHC, there were 4484 DEmRNAs (3264 up‐regulated and 1220 down‐regulated), 273 DEmiRNAs (241 up‐regulated and 32 down‐regulated) and 2564 DElncRNAs (2065 up‐regulated and 499 down‐regulated; Fig. S2). In PAAD, 1951 DEmRNAs (1196 up‐regulated and 755 down‐regulated), 62 DEmiRNAs (47 up‐regulated and 15 down‐regulated) and 463 DElncRNAs (349 up‐regulated and 114 down‐regulated) were screened (Fig. S3). Cross‐cancer analysis showed that a total of 430 up‐regulated DEmRNAs and 76 down‐regulated DEmRNAs, 16 up‐regulated DEmiRNAs and one down‐regulated DEmiRNAs, as well as 107 up‐regulated DElncRNAs and six down‐regulated DElncRNAs, were overlapped among three cohort of aberrantly expressed lnRNAs, miRNAs and mRNAs (Fig. 2A,C,E).

### GSEA of different cohorts

Aiming to explore the enriched gene sets of digestive glands malignances, GSEA was performed in different cancers. The GSEA results showed that 20 gene sets enriched in CHOL, consisting of ‘G2M checkpoint’, ‘E2F targets’ and ‘epithelial mesenchymal transition’, etc., followed by 15 terms in PAAD (‘pancreas beta cells’, ‘estrogen response late’ and ‘glycolysis’, etc.) and 14 terms in LIHC (‘G2M checkpoint’, ‘E2F targets’ and ‘spermatogenesis’, etc.). Furthermore, ‘G2M checkpoint’, ‘E2F targets’ and ‘KRAS down signaling’ were enriched gene sets in CHOL, LIHC and PAAD (Fig. 2B,D,F), respectively, demonstrating the essential role of cell proliferation in digestive gland malignancy.

### Functional enrichment and pathways for overlapped mRNAs

**Fig. 3:**
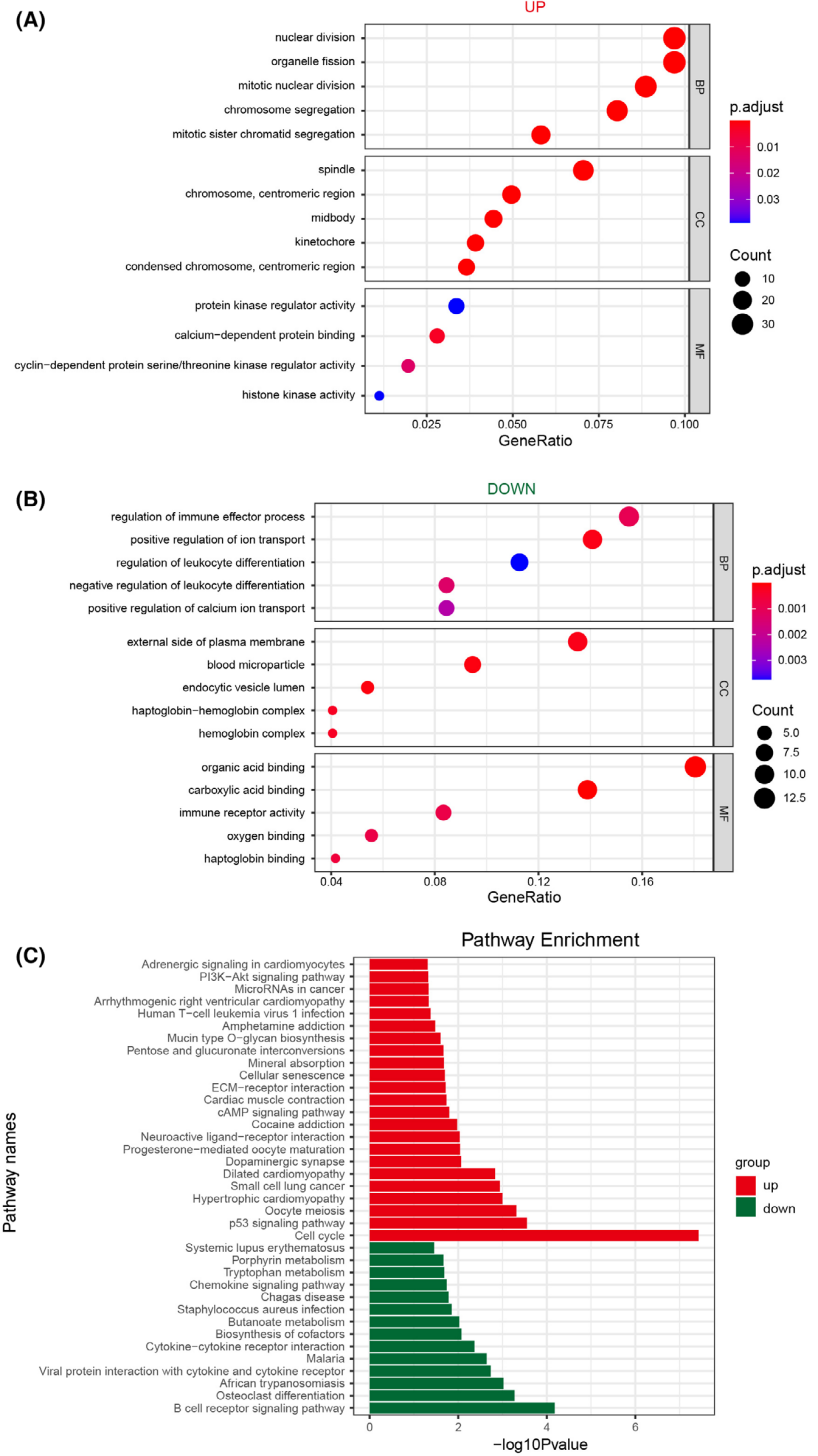
Gene ontology (GO) and Kyoto Encyclopedia of Genes and Genomes (KEGG) analysis in digestive gland malignancies. GO enrichment analysis including biological process (BP), cellular component (CC) and molecular function (MF) for (A) up‐regulated mRNAs and (B) down‐regulated mRNAs. (C) KEGG pathway analysis. Red, up‐regulated genes; green, down‐regulated genes.

Next, we performed GO and KEGG enrichment analyses on the uniformly up‐regulated and down‐regulated DEmRNAs in the three types of tumor to gain insight with respect to the common biological functions of three digestive gland malignancies. Functional annotations of GO enrichment demonstrated that upregulated genes were significantly related with cell division, whereas downregulated genes were significantly associated with transport regulation and immune processes (Fig. 3A,B). The KEGG pathway enrichment analysis indicated that up‐regulated mRNAs were enriched in 20 pathways, such as ‘cell cycle’, ‘p53 signaling pathway’ and ‘microRNAs in cancer’, whereas down‐regulated mRNAs were enriched in 14 pathways, consisting of ‘B cell receptor signaling pathway’, ‘cytokine‐cytokine receptor interaction’ and ‘butanoate metabolism’ (Fig. 3C).

### Construction of the co‐expression network

**Fig. 4:**
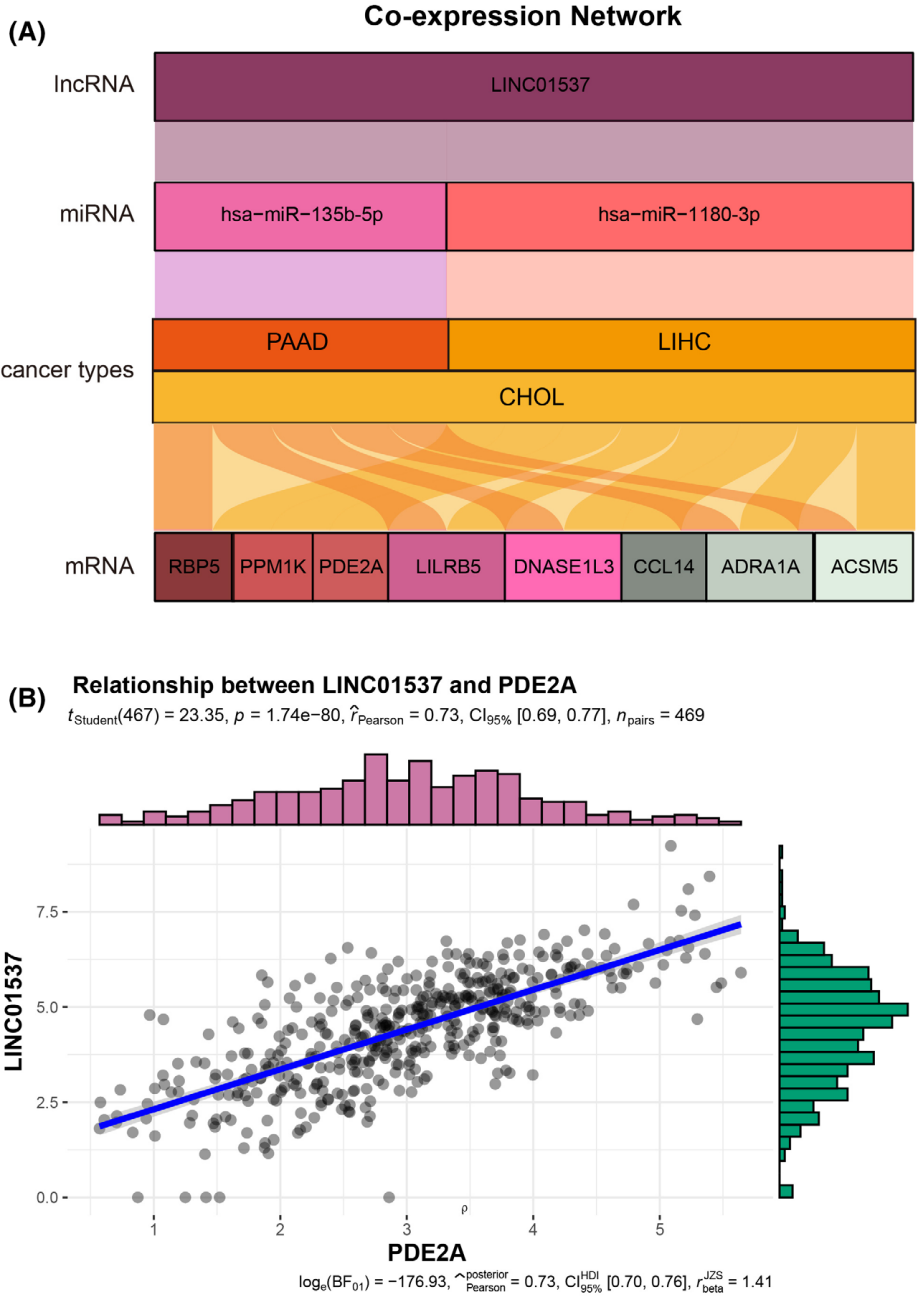
The construction of co‐expression network of digestive gland malignancies. (A) The co‐expression network of digestive gland malignancies. (B) The relationship between LINC01537 and PDE2A.

Given to that ‘microRNAs in cancer’ was enriched in upregulated genes in our analysis, we proposed that a common ceRNA network constructed by mRNAs–miRNA–lncRNAs may be universally involved in the digestive malignancies. To explore the potential ones, we first performed lncRNAs–miRNAs and miRNAs–mRNAs co‐expression analysis in CHOL, LIHC and PAAD, respectively. One hundred and twenty‐two lncRNAs, 19 miRNAs and 580 mRNAs were co‐expressed in the CHOL cohort; 25 lncRNAs, nine miRNAs and 181 mRNAs were co‐expressed in the LIHC cohort; and 28 lncRNAs, nine miRNAs and 245 mRNAs were co‐expressed in the PAAD cohort. We then obtained the intersection of co‐expression networks (Fig. 4A), but no common miRNA profiles were found in the three types of tumors. For example, miR‐1180‐3p participated in LIHC, whereas miR‐135b‐5p was involved in PAAD. Interestingly, miR‐1180‐3p and miR‐135b‐5p both took part in CHOL, suggesting that CHOL may consist of heterogenous populations that are similar to LIHC or PAAD. Although the ceRNA network was unsuccessfully established, we discovered that LINC01537 and eight mRNAs were significantly correlated in all the three types of tumor. The eight mRNAs were acyl‐CoA synthetase medium chain family member 5 (ACSM5), adrenoceptor alpha 1A (ADRA1A), C‐C motif chemokine ligand 14 (CCL14), deoxyribonuclease 1 like 3 (DNASE1L3), leukocyte immunoglobulin like receptor B5 (LILRB5), phosphodiesterase 2A (PDE2A) protein phosphatase, Mg^2+^/Mn^2+^ dependent 1 K (PPM1K) and retinol binding protein 5 (RBP5), among which PDE2A exhibited the strongest correlation with LINC01537 (Fig. 4B and Table S1). Interestingly, PDE2A was a potential target of LINC01537 according to the prediction in the public database starBase v2.0 (http://starbase.sysu.edu.cn). It was suggested that LINC01537 and the eight protein‐coding genes may be interrelated in a miRNA independent manner in hepatic–biliary––pancreatic cancers.

### Paired samples expression analysis and survival analysis of identified significative molecules

**Fig. 5:**
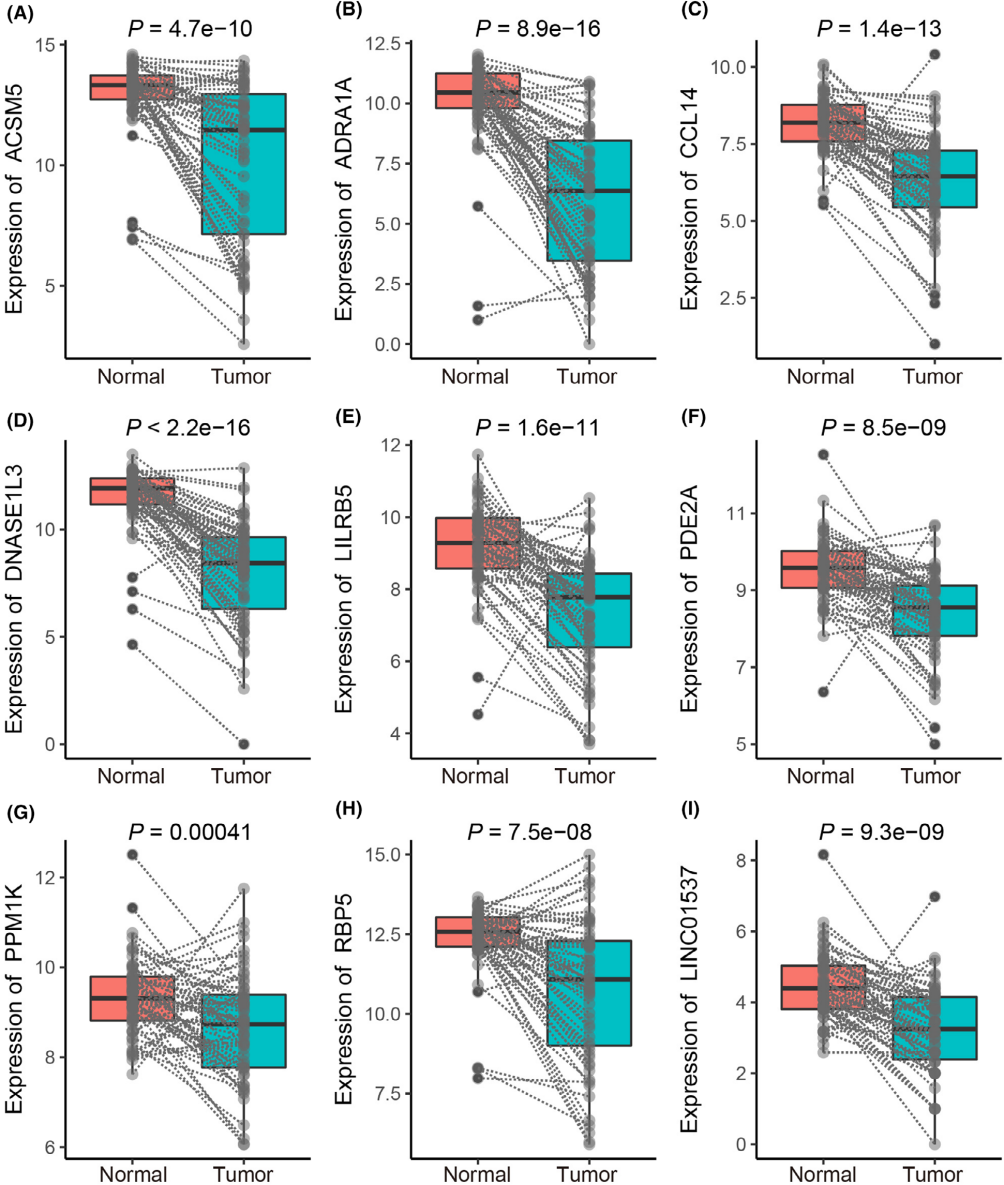
The expression analyses between normal and corresponding tumor tissues in paired samples (62 pairs) in hepatic–biliary–pancreatic tumors. (A) ACSM5, (B) ADRA1A, (C) CCL14, (D) DNASE1L3, (E) LILRB5, (F) PDE2A, (G) PPM1K, (H) RBP5 and (I) LINC01537. Comparisons between groups were performed using a two‐tailed Student's *t* test.

**Fig. 6:**
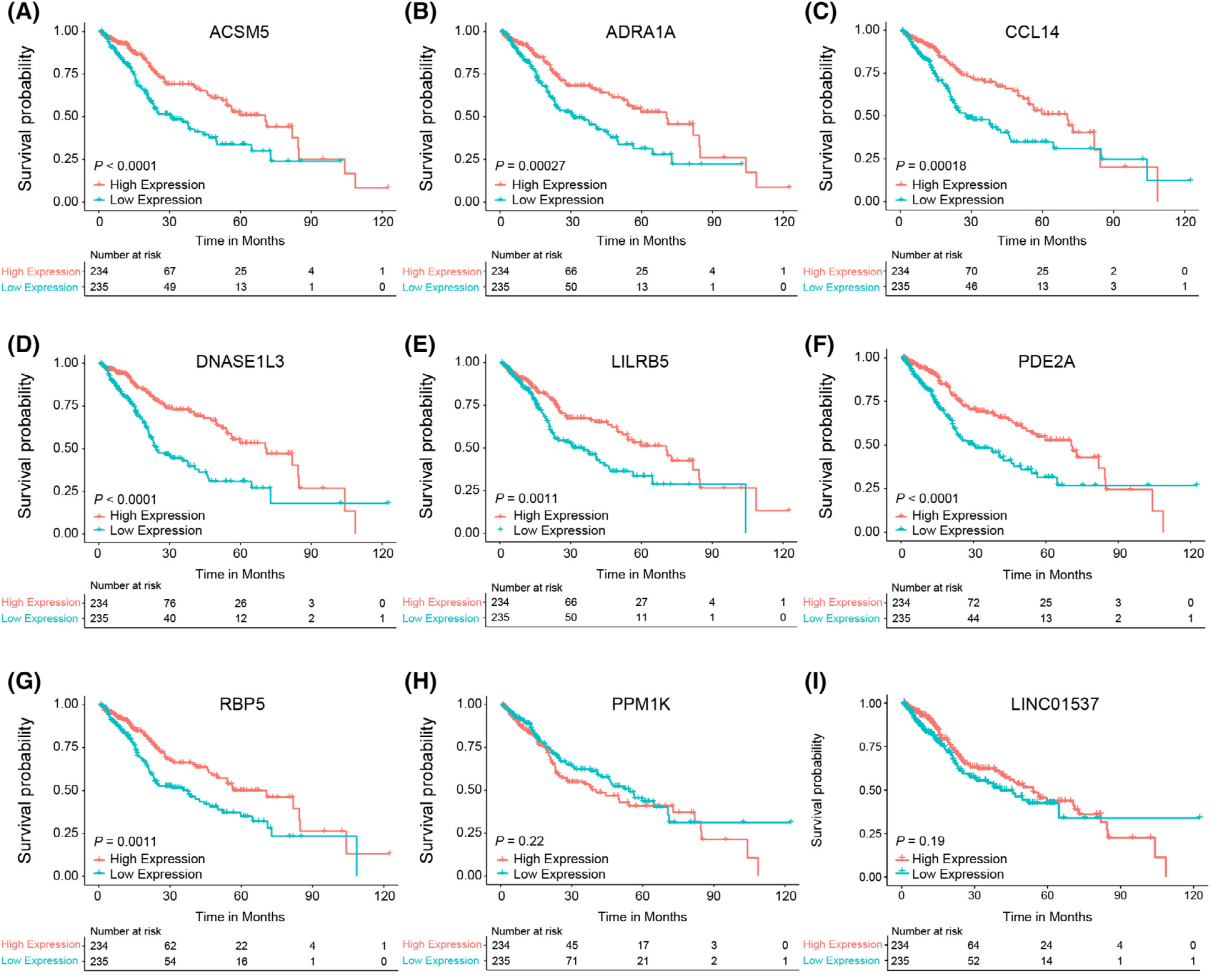
The survival analysis of key molecules in hepatic–biliary–pancreatic cancers. (A) ACSM5, (B) ADRA1A, (C) CCL14, (D) DNASE1L3, (E) LILRB5, (F) PDE2A, (G) RBP5, (H) PPM1K and (I) LINC01537.

To further investigate the importance of the above nine molecules (LINC01537 and eight mRNAs), we performed paired comparison analysis by examining their expression in tumor samples and the corresponding adjacent tissues (62 pairs). The expression of these nine molecules decreased in tumor tissues significantly compared to their matching para‐cancerous tissues (Fig. 5). Moreover, we performed Kaplan–Meier survival analysis to identify the prognostic power of each individual molecule in the OS of patients. Seven mRNAs, including ACSM5, ADRA1A, CCL14, DNASE1L3, LILRB5, PDE2A and RBP5, were linked with a higher OS probability, whereas the contribution of PPM1K and LINC01537 was not significant (Fig. 6). Therefore, we concluded that these seven genes might be protective prognostic factors. Notably, although LINC01537 was not significantly associated with OS in pan‐digestive cancer patients, it was significantly associated with that in hepatic cancer patients (Fig. S4).

### Establishment of the prognostic model

**Fig. 7:**
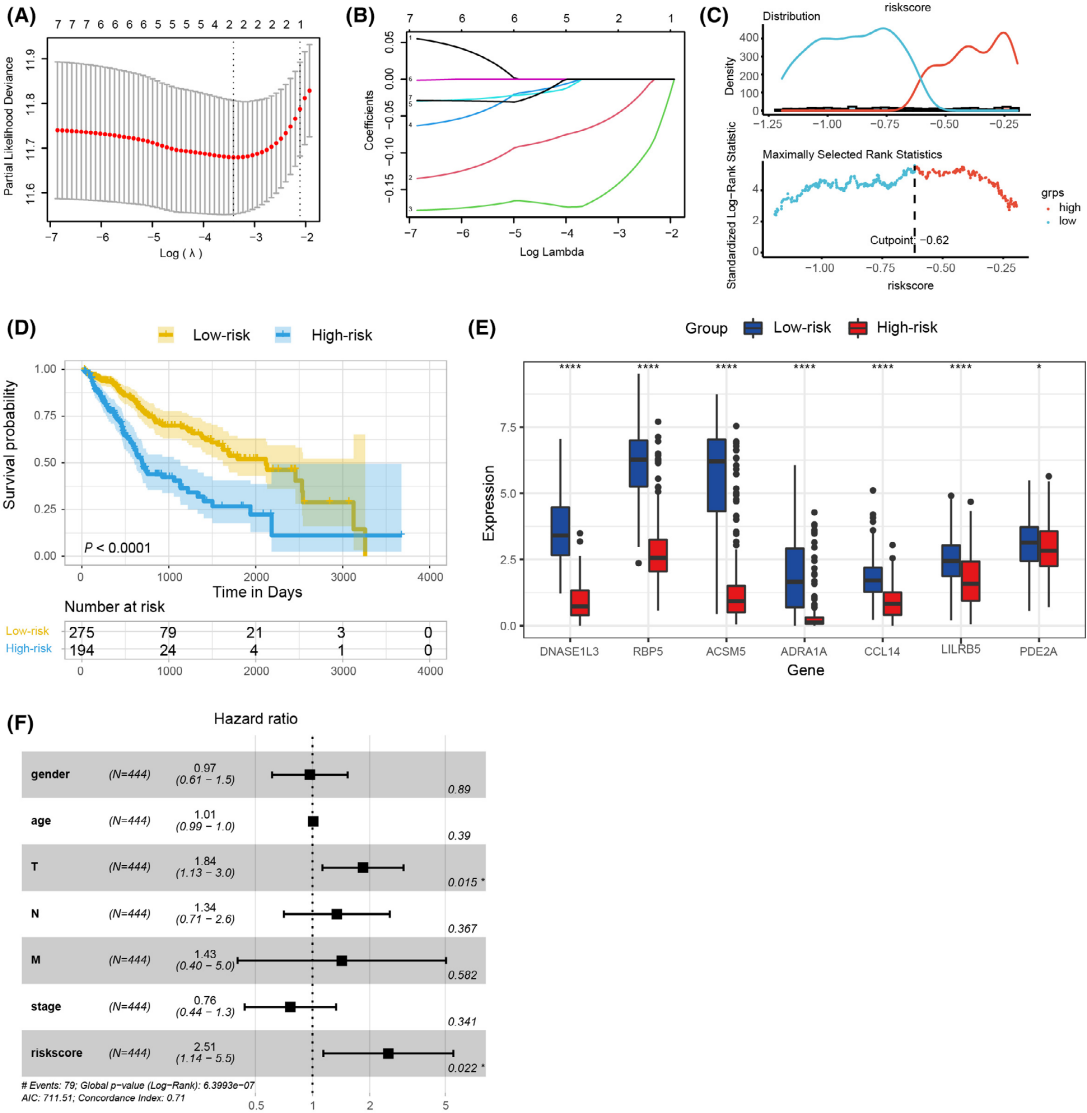
Construction of risk signature in digestive gland malignancies form TCGA cohort. (A) Tuning parameter lambda/λ selection in the LASSO model. The partial likelihood deviance was plotted against log (λ). The dotted vertical lines showing the optimal values through minimum criteria and 1se criteria. (B) Least absolute shrinkage and selection operator (LASSO) coefficient profiles of seven mRNAs selected by univariate cox regression analysis. (C) The optimal cut‐off point value dichotomized risk score into low and high groups. (D) Survival analyses for low‐risk (275 samples) and high‐risk (194 samples) groups using Kaplan–Meier curves (*P* < 0.0001; log‐rank test). (E) The seven key molecules expressed in the low‐risk and high‐risk groups (*P* < 0.0001, *P* < 0.05). (F) Pathologic T and risk score could be regarded as independent prognostic biomarkers using multivariate analyses.

**Fig. 8:**
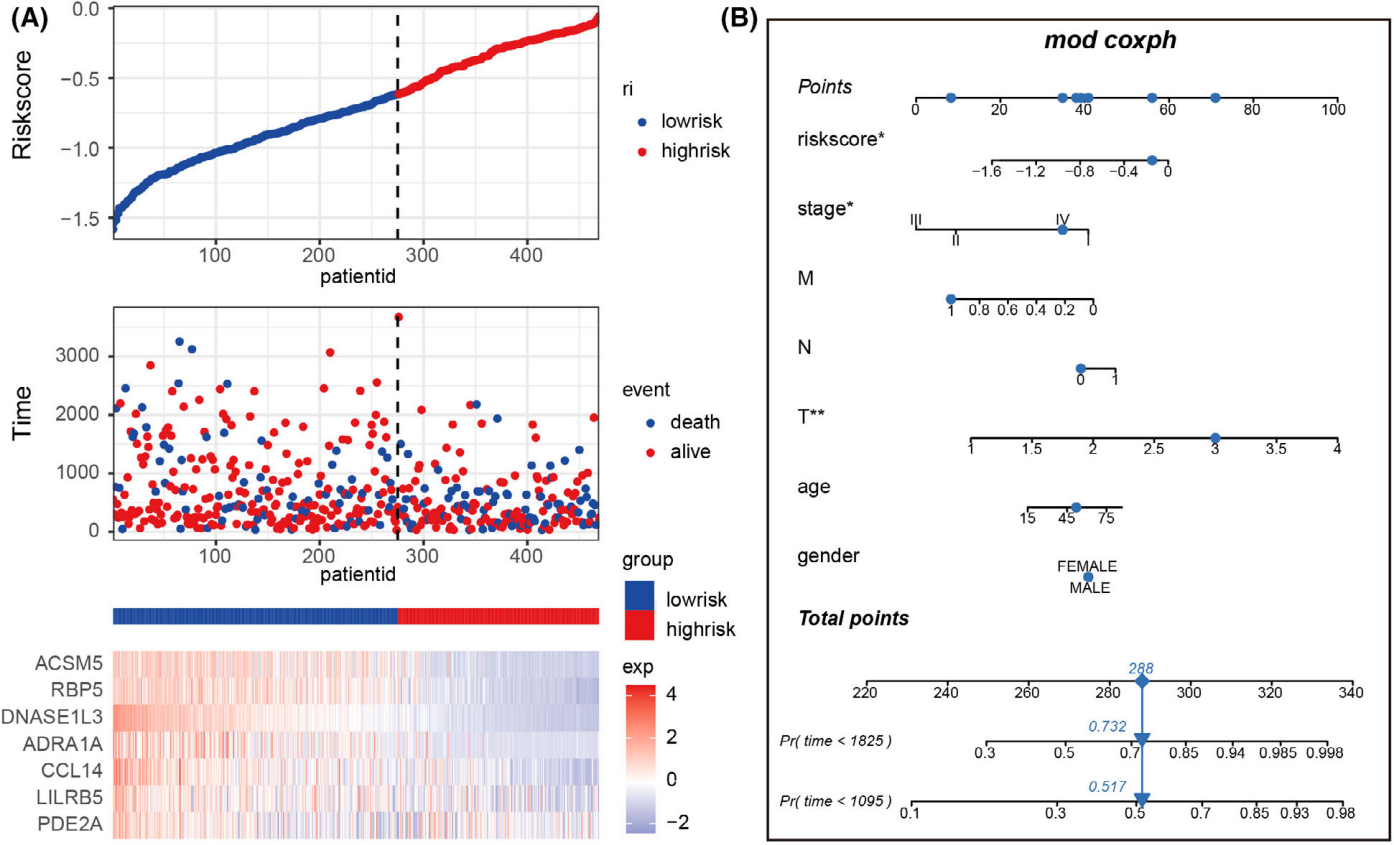
Prognostic value of the risk score gene signature. (A) Hierarchical clustering of seven key genes between low‐risk and high‐risk groups. (B) The nomogram constructed to predict the probability of patient mortality (*P < 0.05, **P < 0.01).

Next, based on the eight molecules, we explored to establish a predictive model that can be universally applied for hepatic–biliary–pancreatic cancer prognosis. Univariate Cox regression analysis was used to select out significant genes contributing to the successful prediction of patients' survival rate. As shown in Table S2, seven mRNAs (ACSM5, ADRA1A, CCL14, DNASE1L3, LILRB5, PDE2A and RBP5) were selected except for PPM1K (*P* = 0.449). Moreover, the hazard ratios of these seven genes were all less than 1, demonstrating that they were protective factors. Considering the potential roles of these seven mRNAs in patient prognosis, we established a LASSO Cox regression model integrating these mRNAs to build a risk score signature (Fig. 7A,B) and screened out two mRNAs (DNASE1L3 and RBP5) by minimizing λ. The two mRNAs strongly represented the risk scores, as shown in Fig. 7C. We then grouped the patients into a low‐risk group with a 5‐year survival rate of approximately 50%, and a high‐risk group with a 5‐year survival rate of approximately 25% based on the cut‐off value −0.62 (Fig. 7D). In total, 5097 DEmRNAs (3337 up‐regulated and 1760 down‐regulated) were identified. As expected, the expression of the seven mRNAs was significantly higher in the low‐risk group than those in the high‐risk group (Fig. 7E). Next, the independent factors associated with patient prognosis, including gender, age, TNM grading and tumor histological staging, were submitted for multivariate Cox regression analysis, respectively. The results of multivariate Cox regression analysis demonstrated that risk score signature could serve as a reliable and independent prognostic biomarker to assess patient outcomes. Furthermore, pathologic T was also significantly related to patient prognosis and could be regarded as the independent prognosis factor as well (Fig. 7F). Patient mortality significantly decreased as the risk values increased (Fig. 8A). The established nomogram, which integrated risk score and independent clinical prognostic factors, could effectively predict the probability of patient mortality (3‐ and 5‐year survival rates) and provide a potential prognostic method in clinics (Fig. 8B).

### External and internal validation of the prediction model

**Fig. 9:**
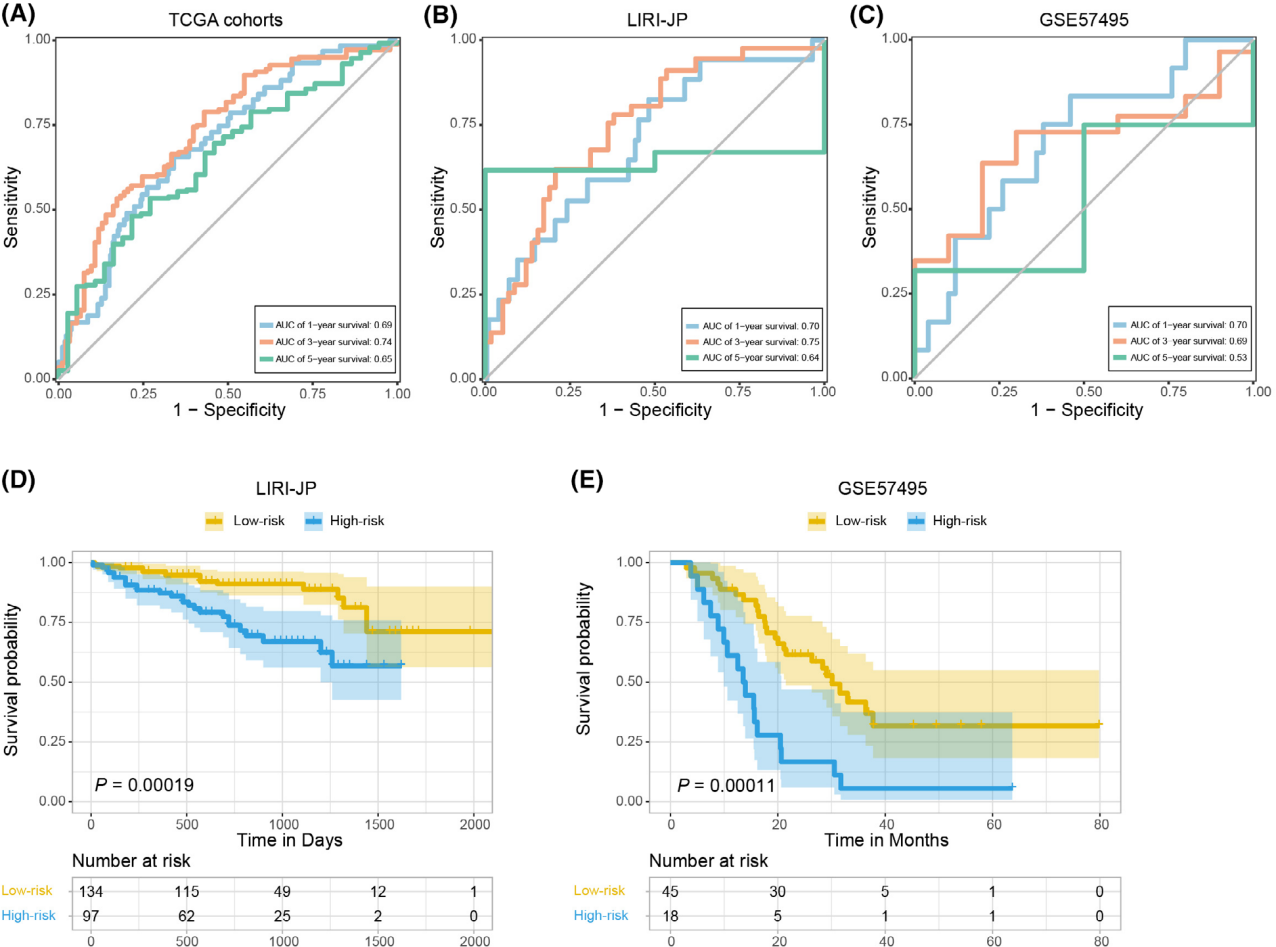
Internal and external validation of the prognostic risk model in digestive gland malignancies. ROC analysis of the prognostic model in the (A) TCGA testing cohorts, (B) LIRI‐JP validation cohort and (C) GSE57495 validation cohort (C). Kaplan–Meier survival analysis of OS between patients with low‐risk scores and high‐risk scores in the (D) LIRI‐JP validation cohort and (E) GSE57495 validation cohort.

**Fig. 10:**
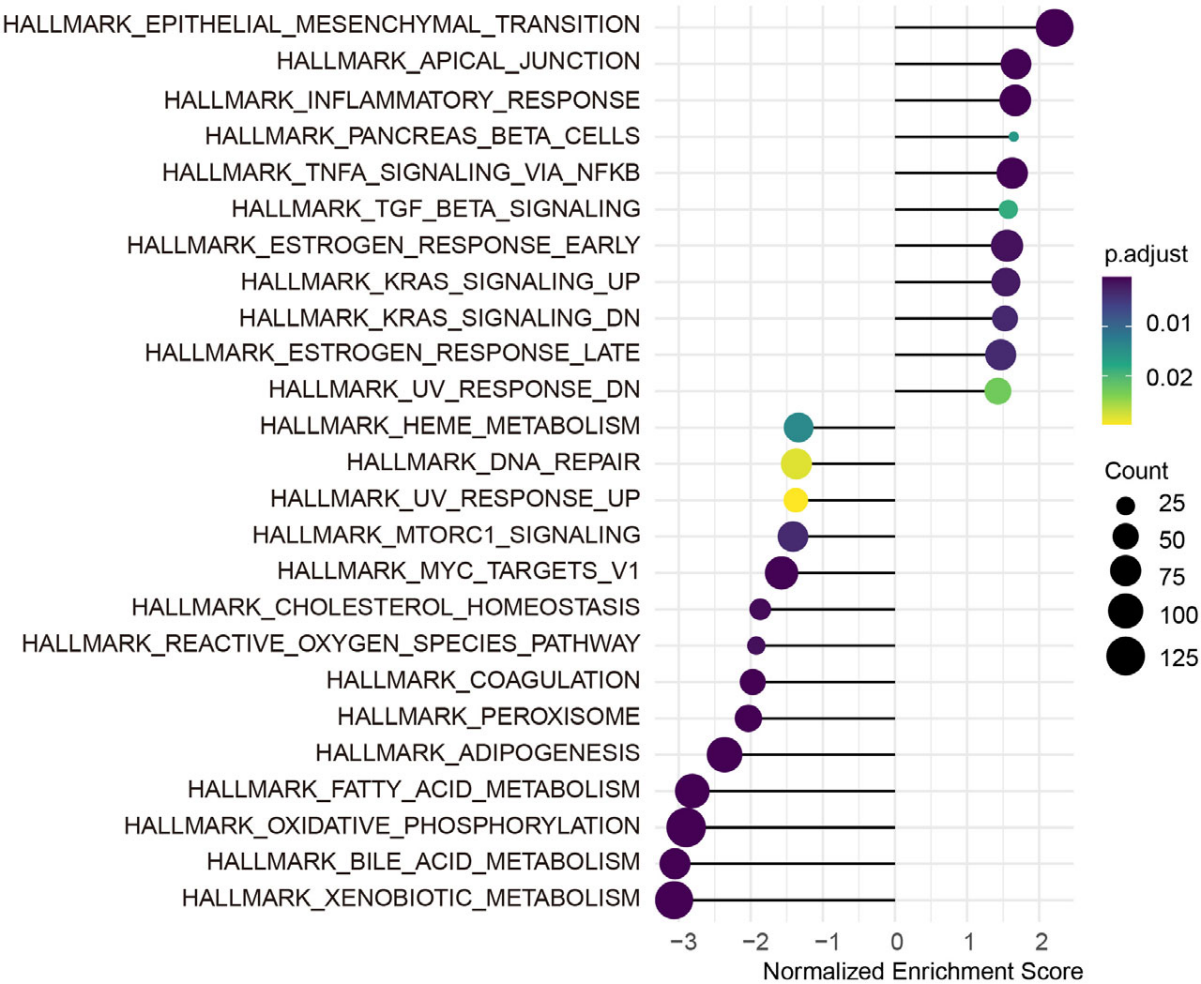
GSEA analysis for mechanism of prognosis in digestive malignancies patients between the high‐risk and low‐risk groups.

For cross‐validation, we applied our prediction model to the internal cohorts (TCGA) and two independent external cohorts (LIRI‐JP and GSE57495). The area under the curve values of the entire TCGA cohorts were 0.69, 0.74 and 0.65 for 1‐, 3‐ and 5‐year survival rates, respectively. (Fig. 9A). Consistently, the area under the curve values were 0.70, 0.75 and 0.64 in the LIRI‐JP cohort and 0.70, 0.69 and 0.53 in the GSE57495 cohort for 1‐, 3‐ and 5‐year survival rates (Fig. 9B,C). Kaplan–Meier analysis also demonstrated that the OS of patients in the low‐risk group was significantly higher than those in the high‐risk group in both external cohorts (Fig. 9D,E). To further investigate the underlying mechanism, GSEA analysis was performed, demonstrating that the differentially expressed genes in the high‐risk group were significantly enriched in the EMT pathway (Fig. 10), suggesting that our prediction model could also predict the EMT occurrence possibly. As shown in Table S3, epithelial marker like E‐cadherin was decreased, whereas mesenchymal markers, including Snail, Twist, vimentin and N‐cadherin, were increased in high‐risk group from hepatic–biliary–pancreatic tumors.

### Mutation landscape of key molecules

Considering their protective roles, we examined the mutation landscape of seven mRNAs in CHOL, LIHC and PAAD, respectively. In CHOL samples, 5.88% of 51 patients experienced mutations in ACSM5, DNASE1L3 and PDE2A (Fig. S5A). In LIHC samples, 2.47% of 364 patients experienced mutations in LILRB5, ACSM5, CCL14 and RBP5 (Fig. S5B). In PAAD samples, 2.25% of 178 patients experienced mutations in ADRA1A, ACSM5, PDE2A, LILRB5 and DNASE1L3 (Fig. S5C). It was found that the ACSM5 exhibited mutation in three tumors. ACSM5, DNASE1L3 and PDE2A showed mutations in CHOL and PAAD. Furthermore, missense mutations are dominant forms.

### PDE2A repressed EMT as a protective factor

**Fig. 11:**
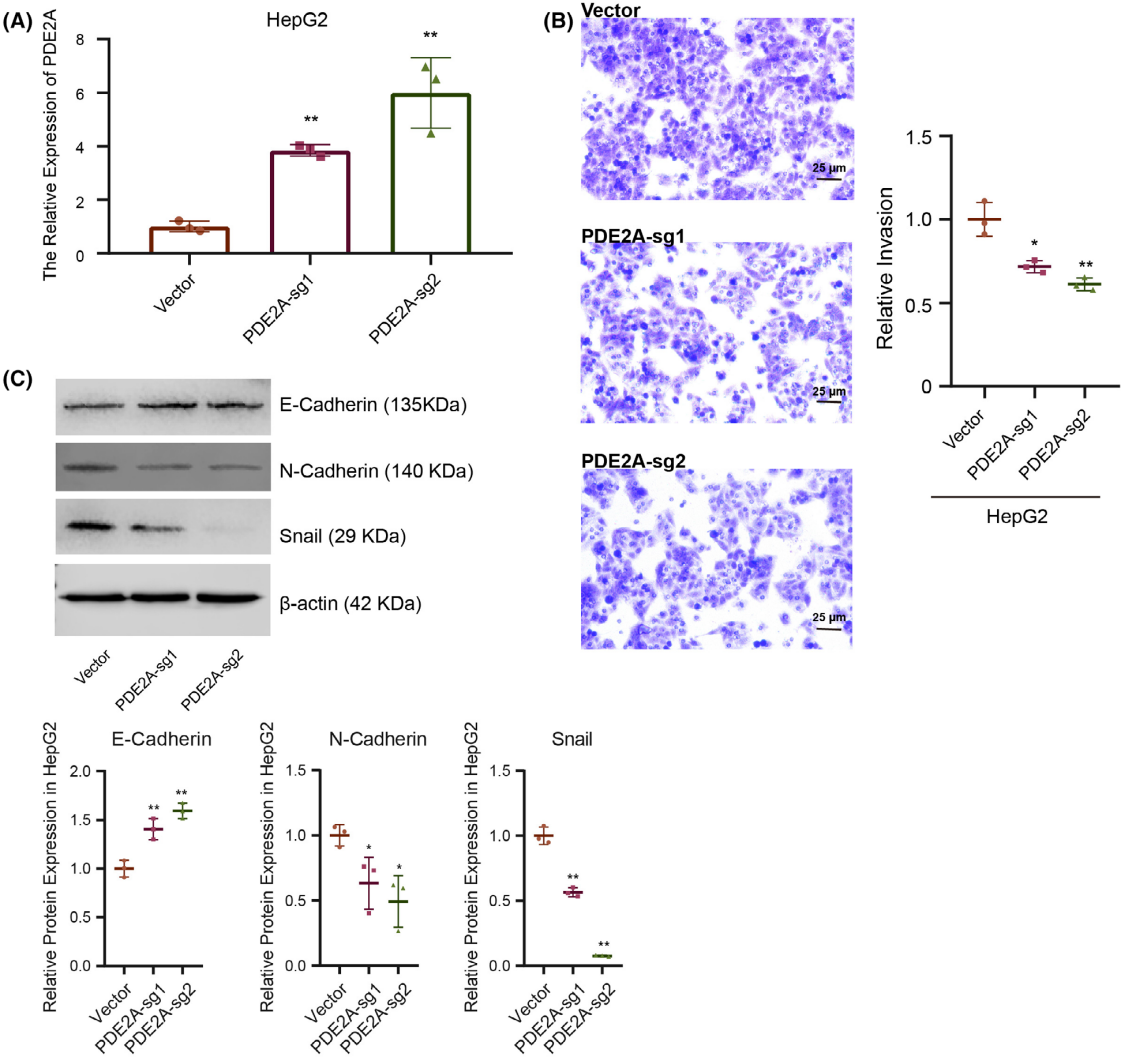
Overexpressed PED2A repressed tumor EMT in LIHC. (A) The relative expression level of PDE2A by a quantitative RT‐PCR (replicates number = 3). (B) Transwell assay (replicates number = 3). Scale bar = 25 μm. (C) Western blot analysis (replicates number = 3). Comparisons between groups were performed using a two‐tailed Student's *t* test. Data are shown as the mean ± SD. *P < 0.05, **P < 0.01.

Given that these genes may be protective factors, we further tested whether they could prevent carcinogenesis of LIHC, the most common cancer in the hepatic–biliary–pancreatic system. We took a widely‐used human liver cancer cell line HepG2 as a model system. Among the seven genes, PDE2A exhibited the lowest expression level in HepG2, as revealed by public RNA‐seq data (Fig. S6). Therefore, we took PDE2A as an example and activated the endogenous expression of PDE2A by dCas9‐VP64 mediated CRSIPRa [33]. The result of a quantitative RT‐PCR indicated that PDE2A was successfully up‐regulated by 4–6‐fold via CRISPRa employing two different sgRNAs (*P* < 0.01; Fig. 11A). Because our analysis suggested that our predictive model distinguished the patients with high and low EMT signatures, we further explored the role of PDE2A in cancer cell invasion and EMT. Transwell assays demonstrated that activation of PDE2A attenuated the invasion ability of HepG2 cells (Fig. 11B). In addition, western blot analysis revealed that epithelial marker E‐cadherin was slightly increased, whereas mesenchymal marker N‐cadherin and EMT‐associated transcription factor Snail were decreased when PDE2A were activated (Fig. 11C). These results suggested that PDE2A was not only a prognostic mark, but also a functional gene that is negatively associated with liver carcinogenesis.

## Discussion

We initially identified the DElncRNAs, DEmiRNAs and DEmRNAs between tumor tissues and para‐cancerous tissues. GSEA analysis indicated that gene sets related to cell cycle and cell division might be the common potential mechanism in digestive malignancies. Functional enrichment analysis demonstrated that GO‐BP terms such as ‘nuclear division’ and ‘organelle fission’ were significantly enriched by upregulated mRNAs, whereas GO‐BP terms such as ‘positive regulation of ion transport’ and ‘regulation of immune effector process’ were significantly enriched by downregulated mRNAs. Moreover, ‘cell cycle’ was significantly enriched by upregulated genes whereas ‘B cell receptor signaling pathway’ was significantly enriched by downregulated genes in digestive gland tumors in KEGG pathway enrichment analysis. Previous studies have demonstrated that cell cycle and cell division are critical for cancer development [34]. B cells, the main effector cells associated with immunity, inhibit tumor progression by secreting immunoglobulins, promoting a T cell response and directly killing cancer cells [35]. Therefore, the dysregulated genes observed in our research might contribute to tumor progression through these signaling pathways.

To better understand the common mechanism of digestive gland malignancies. LINC01537 and eight mRNAs (ACSM5, ADRA1A, CCL14, DNASE1L3, LILRB5, PDE2A, PPM1K and RBP5) were finally screened out according to lncRNAs–miRNAs and miRNAs–mRNAs co‐expression analysis. LINC01537 is located on human chromosome 11q13.4. A previous study has demonstrated that LINC01537 was significantly associated with lung cancer survival, inhibiting tumor proliferation and metastasis, as well as enhanced cellular sensitivity to nilotinib through stabilized PDE2A protein [36]. Another bioinformatic analysis indicated that LINC01537 was a risk factor for patient prognosis and a ferroptosis‐related therapeutic target in lung adenocarcinoma [37]. Therefore, LINC01537 was considered as a biomarker related with survival prediction and therapeutic target in lung cancer. Consistently, LINC01537 was downregulated in digestive gland tumor tissues compared to normal in the present study. Although the correlation between LINC01537 and the survival rate of digestive gland malignancies was not statistically significant (*P* = 0.19), it was significantly correlated with patient prognosis as a protective factor in LIHC (*P* = 0.0045; Fig. S4) from the GEPIA (http://gepia.cancer‐pku.cn) database. The fact that LINC01537 was not significantly correlated with the other two types of cancer might be a result of limited patient samples (LIHC : CHOL : PAAD ≈ 4 : 0.4 : 1.7). However, further functional studies are needed to investigate the specific effects of LINC01537 in carcinogenesis. Survival analysis demonstrated that the high expression of seven mRNAs (ACSM5, ADRA1A, CCL14, DNASE1L3, LILRB5, PDE2A and RBP5) was associated with a significant survival advantage. Therefore, these seven mRNAs, similar to LINC01537, might be tumor suppressors in digestive glands.

ACSM5, as a protein‐coding gene, mainly participates in the cytochrome P450 pathway. GO annotations related to ACSM5 consist of GTP binding and butyrate‐CoA ligase activity [38]. ACSM5 also catalyzes the activation of fatty acids by CoA in the first step of fatty acid metabolism to produce acyl‐CoA [39]. A previous study reported that ACSM5 was identified as a biomarker for lung adenocarcinoma prognosis and was also associated with the tumor microenvironment [38]. However, the detailed roles and specific mechanisms of ACSM5 in tumor progression need further exploration. ADRA1A belongs to the G protein‐coupled receptor superfamily that stimulates the sympathetic nervous system by binding catecholamines. ADRA1A also activates the mitogenic response and regulates the growth and proliferation of many cells [40]. Previous studies have demonstrated that ADRA1A plays a crucial role in various cancer progresses, including hepatocellular carcinoma [41], gastric carcinoma [42], lung cancer [43] and hysterocarcinoma [44]. ADRA1A hypermethylation contributed to liver cancer initiation and was associated with patient prognosis as a promising biomarker for diagnosis [41]. CCL14 is a molecular signal of CC chemokines that induces leukocyte migration during inflammation [45]. CCL14 is a tumor suppressor associated with prognosis in numerous tumors [46]. It was reported that the overexpressed CCL14 suppressed proliferation and promoted apoptosis of hepatocellular carcinoma cells via inhibition of the activation of the Wnt/β‐catenin pathway [45]. DNASE1L3 mediates DNA breakdown during apoptosis. Patients with positive DNASE1L3 expression showed significantly longer overall survival [47]. RBP5 has been shown to be an essential metabolic protein responsible for the storage and transportation of retinol throughout the body. RBP5 is involved in many tumor progressions, including hepatocellular carcinoma [48], cholangiocarcinoma [49], gastric cancer [50] and lung cancer [51]. LILRB5 is a typical type‐1 transmembrane protein, a member of the leukocyte immunoglobulin‐like receptor (LILR) family, containing four extracellular immunoglobulin superfamily domains [52]. LILRB5 expressed in a variety of immune cells from both peripheral blood and the microenvironment in hepatocellular carcinoma patients [53]. PDE2A, as a key member of the phosphodiesterase (i.e. PDE) family, regulates mitochondrial cAMP levels and respiration, participating in multiple physiological activities such as energy metabolism [54]. The genomic location of the gene for PDE2A is adjacent to LINC01537. Thus it is possible that a physical interaction between PDE2A and LINC01537 exists, which has been observed in lung cancer. Gong *et al*. [36] reported that LINC01537 promoted PDE2A expression via RNA–RNA interaction to stabilize PDE2A mRNA, thus eliciting PDE2A effects on the energy metabolism of tumors, including both the Warburg effect and mitochondrial respiration. Consistently, LINC01537 also exhibited significant correlation with PDE2A in our analysis (*r* = 0.73, *P* = 1.74 × 10^–80^). Therefore, we propose that LINC01537/PDE2A might repress the development of digestive gland malignancies as well, such as tumor proliferation, invasion and metastasis.

Based on these seven genes, we constructed a predictive model to evaluate the risk of patients’ malignancy. Multivariate Cox regression analysis demonstrated that both pathologic T and risk score were independent prognostic factors. In addition, the nomogram model composed of pathologic T and the risk group accurately predicted the malignancies, which might be helpful in clinics with respect to guiding the clinical practice of cancer prognosis. GSEA analysis further indicated that EMT might be a potential mechanism of poor outcome in the high‐risk group of patients. EMT is a critical step in cancer metastasis. Metastasis is the leading cause of cancer‐associated deaths [55]. During this process, epithelial markers such as E‐cadherin and claudin are decreased, whereas mesenchymal markers such as N‐cadherin and vimentin increased [56]. The switch of these markers was controlled by transcription factors, such as Snail, Slug and Twist [57]. Thus, our model may also be useful for estimating EMT in patients of all samples combined from hepatic–biliary–pancreatic cancers. The same result was observed and validated in liver cancer. Consistently, we demonstrated that PDE2A activation enhanced the expression of epithelial marker E‐cadherin, whereas it reduced the expression of mesenchymal marker N‐cadherin and transcription factor Snail in HepG2 cells. Therefore, the genes in our model may be functionally involved in the occurrence of EMT.

In the present study, we identified risk score, comprising the expression of seven genes (ACSM5, ADRA1A, CCL14, DNASE1L3, LILRB5, PDE2A and RBP5), and pathologic T, as robust and independent universal biomarkers of digestive gland malignancies. Our nomogram model based on these two independent prognostic factors could efficiently predict the prognosis of patients and probably also the occurrence of EMT. However, the clinical application of this nomogram model and the exact molecular mechanism require further research in digestive gland malignancies. The mechanism regarding the key molecules also needs further study, especially for LINC01537. We also found that PDE2A might play a role in the metastasis of hepatocellular carcinoma. In conclusion, the present study demonstrates a shared molecular mechanism underlying hepatic, biliary and pancreatic cancers. Meanwhile, it also improves our cognition of the heterogeneity and complexity in digestive gland malignancies, which require more effective diagnostic, prognostic and therapeutic approaches.

## Conflict of interest

The authors declare no conflict of interest.

## Author contributions

*Design*: JZ and CCW. *Data collection and analysis*: JZ, JX, YXW, XZ and JJC. *Data interpretation*: JZ. *Manuscript writing*: JZ and CCW. *Final approval of manuscript*: All authors. The first draft of the manuscript was written by JZ and all authors commented on previous versions of the manuscript. All authors have read and approved the final version of the manuscript submitted for publication.

## Supporting information


**Fig. S1.** Differences between normal and CHOL samples. Principal component analysis for lnRNAs (A), miRNAs (B) and mRNAs (C). The heatmap of differentially expressed lnRNAs (D), miRNAs (E) and mRNAs (F). The volcano plot of differentially expressed lnRNAs (G), miRNAs (H) and mRNAs (I).
**Fig. S2.** Differences between normal and LIHC samples. The heatmap of differentially expressed lnRNAs (A), miRNAs (B) and mRNAs (C). The volcano plot of differentially expressed lnRNAs (D), miRNAs (E) and mRNAs (F).
**Fig. S3.** Volcano plots of differentially expressed lnRNAs (A), miRNAs (B) and mRNAs (C) between normal and PAAD samples.
**Fig. S4.** Survival analysis of LINC01537 in the TCGA‐LIHC cohort.
**Fig. S5.** Mutation landscape of key molecules associated with patient prognosis in digestive gland malignancies. (A) TCGA‐CHOL cohort. (B) TCGA‐LIHC cohort. (C) TCGA‐PAAD cohort.
**Fig. S6.** Key molecules expression levels in HepG2 cell line.

**Table S1.** The correlation between LINC01537 and eight mRNAs.
**Table S2.** The significant genes contributing to patient prognosis by univariate Cox regression analysis.
**Table S3.** The differentially expressed EMT markers between the high‐risk group and low‐risk group.

## Data Availability

The data that support these findings in this study are openly available in GDC Data Portal (https://portal.gdc.cancer.gov), ICGC Data Portal (https://dcc.icgc.org) and NCBI's Gene Expression Omnibus. The data from GEO are accessible through https://www.ncbi.nlm.nih.gov/geo/query/acc.cgi?acc=[GSE57495] and https://www.ncbi.nlm.nih.gov/geo/query/acc.cgi?acc=[GSE5203], GEO Series accession numbers [GSE57495] and [GSE5203]. All of the data are publicly available and there are no ethical issues involved.
