# Supplementary material for: A universal co‐expression gene network and prognostic model for hepatic–biliary–pancreatic cancers identified by integrative analyses
Source: FEBS Open Bio. 2022 Sep 23;12(11):2006–24. doi: 10.1002/2211-5463.13478 (PMC9623511; doi:10.1002/2211-5463.13478)
Supplement: Supplementary file 1 — Fig. S1. Differences between normal and CHOL samples. Principal component analysis for lnRNAs (A), miRNAs (B) and mRNAs (C). The heatmap of differentially expressed lnRNAs (D), miRNAs (E) and mRNAs (F). The volcano plot of differentially expressed lnRNAs (G), miRNAs (H) and mRNAs (I). Fig. S2. Differences between normal and LIHC samples. The heatmap of differentially expressed lnRNAs (A), miRNAs (B) and mRNAs (C). The volcano plot of differentially expressed lnRNAs (D), miRNAs (E) and mRNAs (F). Fig. S3. Volcano plots of differentially expressed lnRNAs (A), miRNAs (B) and mRNAs (C) between normal and PAAD samples. Fig. S4. Survival analysis of LINC01537 in the TCGA‐LIHC cohort. Fig. S5. Mutation landscape of key molecules associated with patient prognosis in digestive gland malignancies. (A) TCGA‐CHOL cohort. (B) TCGA‐LIHC cohort. (C) TCGA‐PAAD cohort. Fig. S6. Key molecules expression levels in HepG2 cell line. [file FEB4-12-2006-s001.docx]

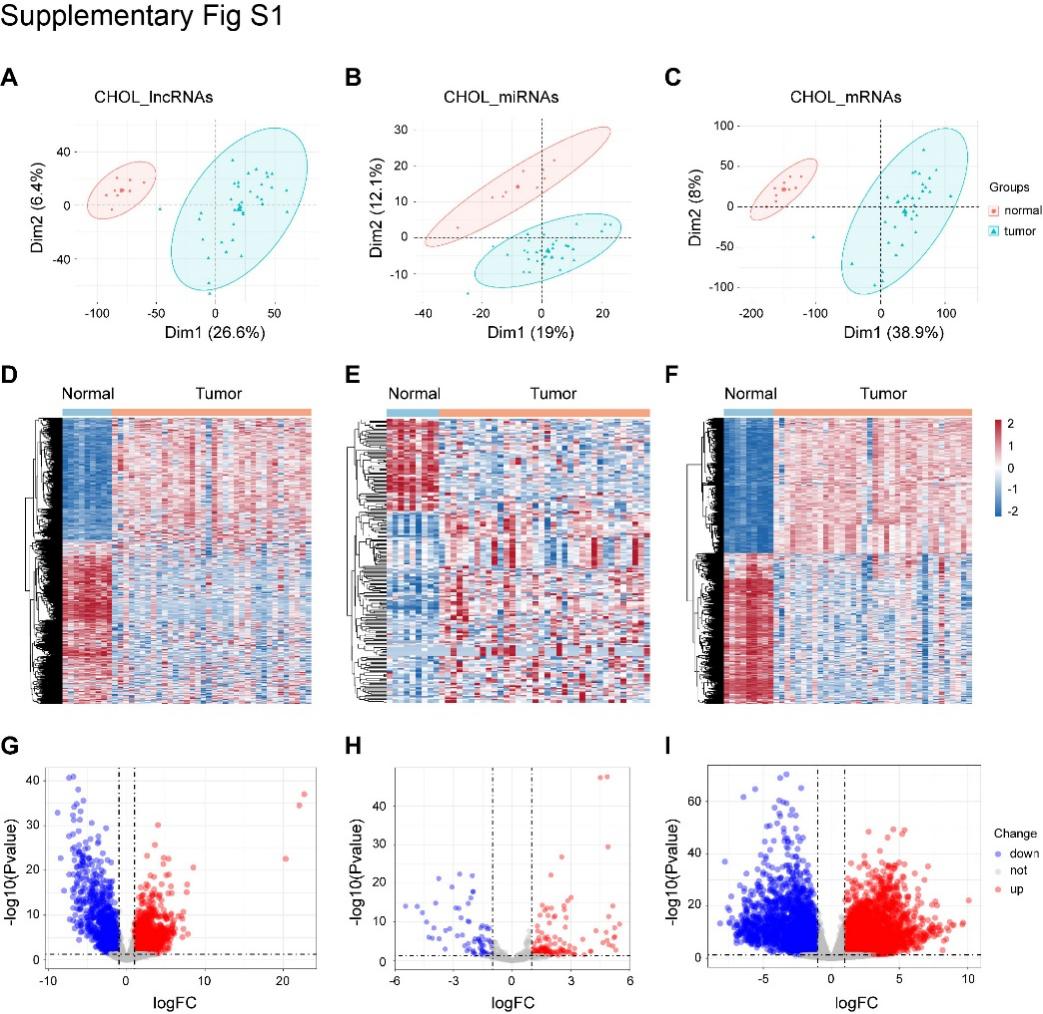


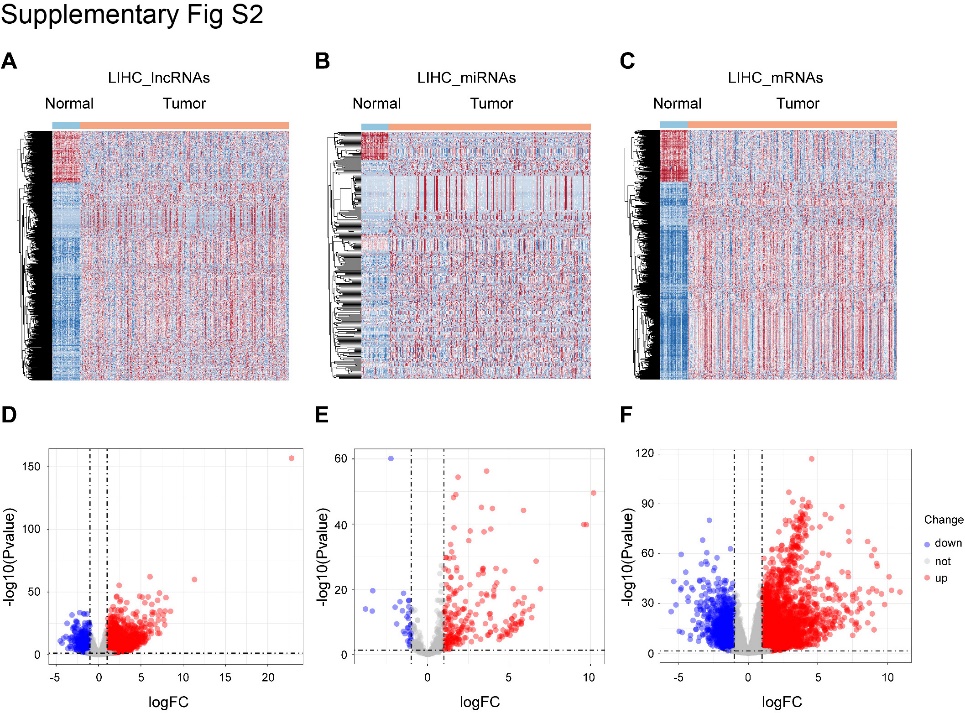


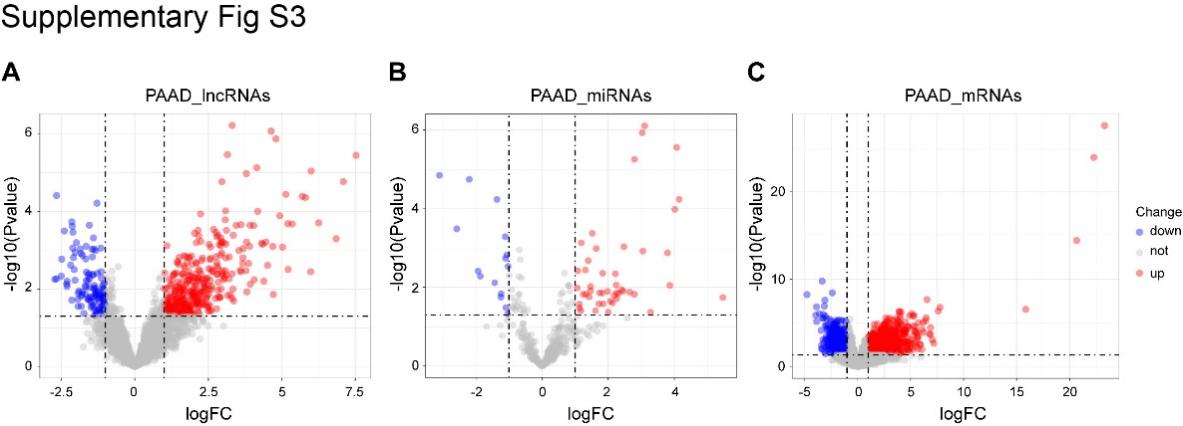


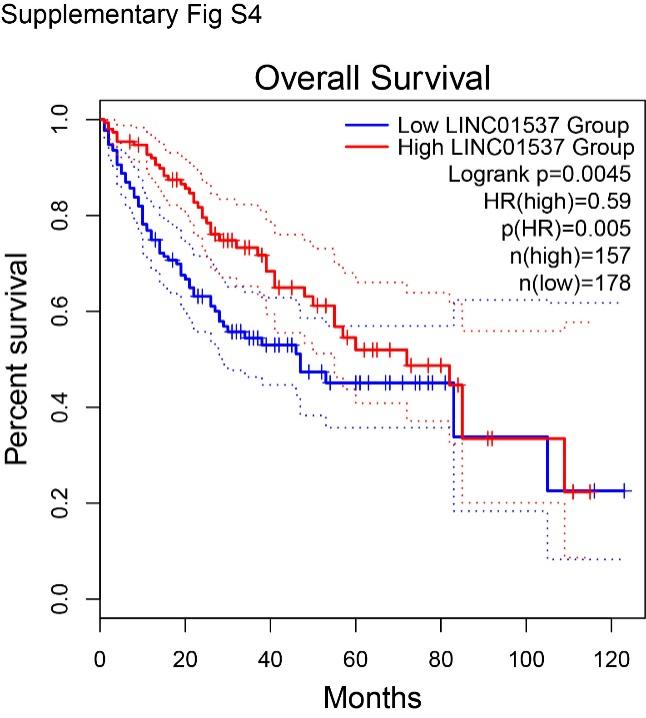


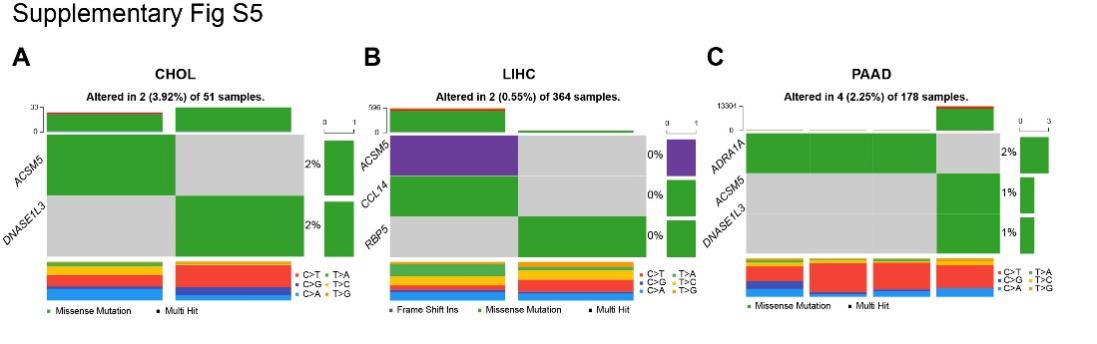


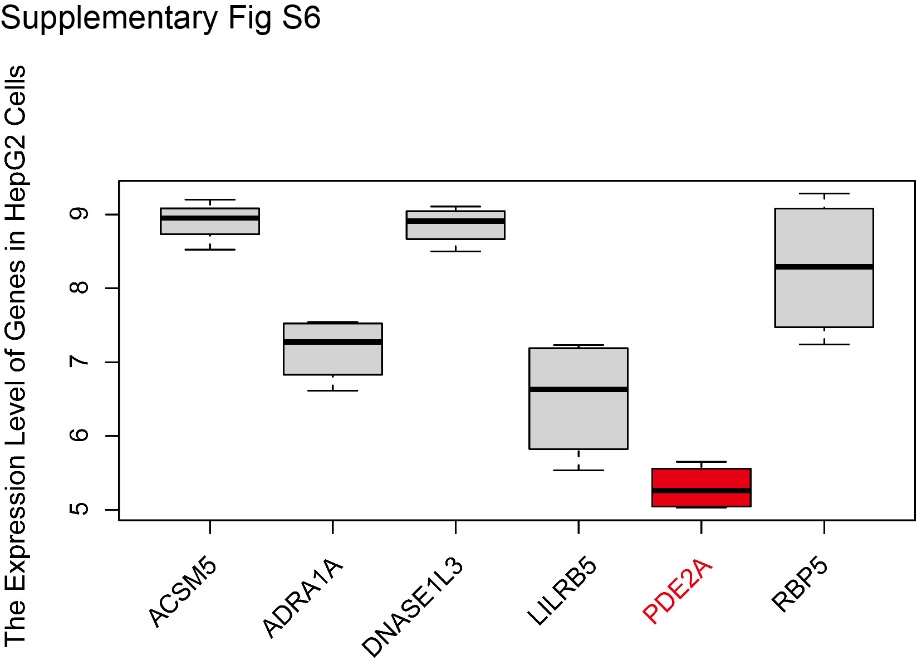


**Supplementary Fig S1** Differences between normal and CHOL samples. Principal component analysis for lnRNAs (A), miRNAs (B) and mRNAs (C). The heatmap of differentially expressed lnRNAs (D), miRNAs (E) and mRNAs (F). The volcano plot of differentially expressed lnRNAs (G), miRNAs (H) and mRNAs (I).

**Supplementary Fig S2** Differences between normal and LIHC samples. The heatmap of differentially expressed lnRNAs (A), miRNAs (B) and mRNAs (C). The volcano plot of differentially expressed lnRNAs (D), miRNAs (E) and mRNAs (F).

**Supplementary Fig S3** Volcano plots of differentially expressed lnRNAs (A), miRNAs (B) and mRNAs (C) between normal and PAAD samples.

**Supplementary Fig S4** Survival analysis of LINC01537 in TCGA-LIHC cohort.

**Supplementary Fig S5** Mutation landscape of key molecules associated with patient prognosis in digestive gland malignancies. (A) TCGA-CHOL cohort. (B) TCGA-LIHC cohort. (C) TCGA-PAAD cohort.

**Supplementary Fig S6** Key molecules expression levels in HepG2 cell line.
