# Supplementary material for: A universal co‐expression gene network and prognostic model for hepatic–biliary–pancreatic cancers identified by integrative analyses
Source: FEBS Open Bio. 2022 Sep 23;12(11):2006–24. doi: 10.1002/2211-5463.13478 (PMC9623511; doi:10.1002/2211-5463.13478)
Supplement: Supplementary file 2 — Table S1. The correlation between LINC01537 and eight mRNAs. Table S2. The significant genes contributing to patient prognosis by univariate Cox regression analysis. Table S3. The differentially expressed EMT markers between the high‐risk group and low‐risk group. [file FEB4-12-2006-s002.docx]

**Supplementary Table 1.** The correlation between LINC01537 and eight mRNAs

| **Gene Names** | **Correlation** | ***P* value** |
| --- | --- | --- |
| LINC01537 | 1 | 0 |
| ACSM5 | 0.203535934111454 | 8.88438868275953E-06 |
| RBP5 | 0.17146742172844 | 0.000190624409901194 |
| LILRB5 | 0.381276856877677 | 1.12325136363592E-17 |
| DNASE1L3 | 0.475813517621415 | 7.16627444628452E-28 |
| ADRA1A | 0.225615898327932 | 7.94207137106747E-07 |
| PPM1K | 0.192689678762808 | 0.000026542636744654 |
| CCL14 | 0.505451801811377 | 8.7828578738702E-32 |
| PDE2A | 0.733958848725291 | 1.74459154051542E-80 |

**Supplementary Table 2.** The significant genes contributing to patient prognosis by univariate Cox regression analysis

| **Gene Names** | **Coef** | ***P* value** | **HR** |
| --- | --- | --- | --- |
| ACSM5 | -0.122 | < 0.01 | 0.885 |
| RBP5 | -0.191 | < 0.01 | 0.826 |
| LILRB5 | -0.278 | < 0.01 | 0.757 |
| DNASE1L3 | -0.273 | < 0.01 | 0.761 |
| ADRA1A | -0.225 | < 0.01 | 0.799 |
| PPM1K | 0.049 | 0.449 | 1.05 |
| CCL14 | -0.427 | < 0.01 | 0.652 |
| PDE2A | -0.175 | 0.023 | 0.839 |

**Abbreviations: HR, Hazard Ratio**

**Supplementary Table 3.** The differentially expressed EMT markers between high-risk group and low-risk group

| **Genes Names** | **log2FoldChange** | **pvalue** | **padj** | **change** |
| --- | --- | --- | --- | --- |
| SNAI1 (Snail) | 0.9472943 | 3.646356e-14 | 8.616137e-14 | up |
| SNAI2 (Slug) | 0.3094899 | 0.02068828 | 0.02567461 | not |
| TWIST1 (Twist) | 3.546844 | 6.601633e-84 | 3.535359e-82 | up |
| VIM (vimentin) | 1.123914 | 1.142773e-32 | 5.838808e-32 | up |
| CDH1 (E-cadherin) | -1.195006 | 3.96171e-19 | 1.147007e-18 | down |
| CDH2 (N-cadherin) | 0.6899756 | 4.432221e-12 | 9.551265e-12 | up |
